# Defective HIV DNA genomes provide ancestral relevance critical for phylogenetic inference of reservoir dynamics

**DOI:** 10.1101/2025.10.03.680205

**Published:** 2025-11-14

**Authors:** Lauren E Droske, Andrea S Ramirez-Mata, Melanie N Cash, Jose Estrada, Stephen D Shank, Adam Browning, Faezeh Rafiei, Sergei L Kosakovsky Pond, Marco Salemi, Brittany Rife Magalis

**Affiliations:** a University of Florida, Department of Pathology, Immunology, and Laboratory Medicine, Gainesville, 32610, USA; b University of Florida, Emerging Pathogens Institute, Gainesville, 32610, USA; c Temple University, Department of Biology, Philadelphia, 19122, USA; d Temple University, Institute for Genomics and Evolutionary Medicine, Philadelphia, 19122, USA; e University of Louisville, Department of Biochemistry and Molecular Genetics, Louisville, KY, 40202, USA

The authors have withdrawn this manuscript due to a duplicate posting of manuscript number BIORXIV/2022/490630. Therefore, the authors do not wish this work to be cited as reference for the project. If you have any questions, please contact the corresponding author. The correct preprint can be found at doi: 10.1101/2022.05.04.490630.

